# Preterm Delivery and Psycho–Social Determinants of Health Based on World Health Organization Model in Iran: A Narrative Review

**DOI:** 10.5539/gjhs.v5n1p52

**Published:** 2012-11-04

**Authors:** Mahrokh Dolatian, Arash Mirabzadeh, Ameneh Setareh Forouzan, Homeira Sajjadi, Hamid Alavi Majd, Farnoosh Moafi

**Affiliations:** 1 Social Determinant of Health Research Center, University of Social Welfare and Rehabilitation Sciences, Tehran, Iran; 2 Department of Psychiatric, University of Social Welfare and Rehabilitation Sciences, Tehran, Iran; 3 Department of Biostatistics, School of Paramedical Sciences, Shahid Beheshti University of Medical Sciences, Tehran, Iran; 4 Department of Midwifery, Shahid Beheshti University of Medical Sciences, Tehran, Iran

**Keywords:** preterm delivery, pregnancy outcomes, prevalence, socioeconomic condition, structural determinant, Intermediary determinants, psychosocial factor, behavioral factor and Maternal circumstance, health system

## Abstract

**Background:**

Preterm delivery is still the primary cause of mortality and morbidity in infants, which shows a problematic condition in the care of pregnant women all over the world. This review study describes prevalence and psycho - socio-demographic as well as obstetrical risk factors related to live preterm delivery (PTD) in the recent decade in Iran.

**Methods:**

A narrative review was performed in Persian and international databases including PubMed, SID, Google Scholar, Iran Medex, Magiran and Irandoc from 2001 to 2010 with following keywords: preterm delivery and pregnancy outcomes with (prevalence, socioeconomic condition, structural determinant, Intermediary determinants, Psychosocial factor, Behavioral factor and Maternal circumstance, Health system) All of article was reviewed then categorized based on WHO model.

**Results:**

Totally 52 article were reviewed and 35 articles were selected, of which 26 were cross-sectional or longitudinal, 9 were analytical (cohort or case-control). The prevalence rates of preterm delivery in different cities of Iran were reported between 5.6% in Quom to 39.4% in Kerman. The most common social factors in structural determinant were educational level of mother, and in intermediary determinants were Psychosocial factor (maternal anxiety and stress during pregnancy), Behavioral factor and Maternal circumstance (violation and trauma) and in Health system, lack of prenatal care.

**Conclusion:**

The prevalence rate of preterm delivery is a matter of concern. Since many psycho-social factors may affect on the condition and its high rate in poor communities might reveals a causal relationship among biological and psychosocial factors, performing etiological investigations is recommended.

## 1. Introduction

Preterm delivery, which is defined as childbirth prior to 37 weeks of gestation, is a major determinant of neonatal mortality and morbidity with long-term implications for health (Beck et al., 2010). Complications related to preterm delivery can place a heavy burden on limited health resources. Premature newborns are more likely to have cerebral palsy, severe brain injury, retinopathy, necrotizing enterocolitis, and respiratory disorders. The risk of motor sensory problems, learning disabilities, and behavioral complications is increased in children born prematurely compared with normal children (Medicine, 2007).

No global statistics are available on the prevalence of preterm delivery. Current figures from developed countries such as the United States, United Kingdom, and Scandinavia show an increase in the number of preterm delivery in the last 20 years (Beck et al., 2010). The prevalence of preterm delivery has been reported as 5% in developed countries and 25% in developing countries (Johnson et al., 2009; Steer, 2005). In Iran, the prevalence has been reported to be between 5.6% and 13.4% (Kamali Fard M, 2010).

Many unknown and related factors contribute to the development of preterm delivery (Behrman and Butler, 2007). This syndrome may be triggered by many mechanisms (Berghella et al., 2007). Several social factors influence the preterm delivery (Cunningham et al., 2010), namely biological and genetic factors, maternal or fetal medical condition, history of premature birth or stillborn death, behavioral problems, cigarette smoking, low socioeconomic conditions, multiple pregnancy, lack of maternal weight gain during pregnancy, drug abuse, inappropriate family planning, poor antenatal care, absence of spouse, misconduct, emotional stress, environmental factors, and inappropriate behaviors (Goldenberg et al., 2008; Beck et al., 2010, Cunningham et al., 2010; EdwinChandraharan, 2005). The high prevalence of preterm delivery in the poor suggests an etiological relationship between this condition and bio-psychosocial factors. In today’s world, health perspectives are extended to include social determinants. By itself or by influencing on one another, each of these determinants greatly affects on health condition (Health WCoSDo, 2008).

**Figure 1:**
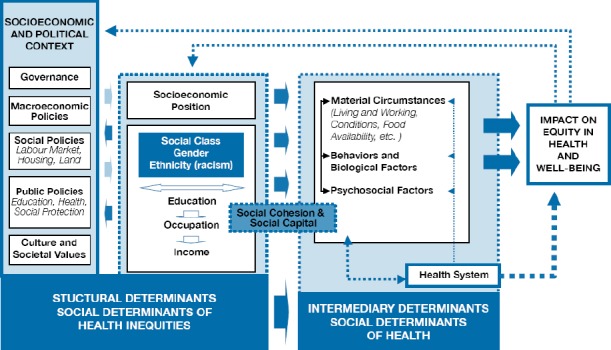
Final form of the CSDH conceptual framework (Solar & Irwin, 2010)

According to the WHO conceptual framework of Commission on Social Determinants affecting on health (Figure 1), they include: 1. Structural determinants in 2 categories: (a) Socioeconomic and political context such as political bodies as well as economic processes, culture, and the function of social welfare system and (b) Other structural determinants such as education, income, sex, race, ethnicity and employment status, which create different unequal socioeconomic groups and, ultimately, form the social class of a person; 2. Intermediary determinants of health in the middle part of the model. In fact, the WHO model implies that structural determinants do not have a direct influence on people’s health but exert their effects through intermediary determinants. These determinants create variations in susceptibility and exposure to conditions that threaten health, including living conditions, work conditions, accessibility to food and health services, psychosocial factors (e.g., psychosocial stress), behavioral factors (e.g., smoking and alcohol consumption), lifestyle and social support, social norms and barriers in choosing healthy life manner. The model indicates that people in lower socioeconomic groups tend to exhibit more harmful health habits than those in higher socioeconomic groups (Solar & Irwin, 2010).

Studies in developed countries have investigated the role of many structural determinants in the development of preterm delivery, such as socioeconomic position, education, occupation, and income. These studies reveal that the lower the socioeconomic condition, the higher the rate of preterm delivery. They suggest those inappropriate health behaviors, lack of prenatal care, insufficient nutrition, anemia, drug abuse, and other maternal conditions increase under poor socioeconomic conditions. These factors have been associated with higher rates of abortion, obstetrical complications, stillbirth, and preterm delivery. Significant relationships have also been identified between preterm delivery and inappropriate housing, low maternal education, and income level (Minkler et al., 2006; Taylor-Robinson et al., 2011; Li et al., 2010; Vettore et al., 2010).

In addition, many studies have been conducted on intermediary determinants of the development of preterm delivery. According to recent reports, preterm delivery is related to stressful life events, anxiety, depression, job stress, and physical abuse (Blumenshine et al., 2010, Kramer et al., 2001). Another intermediary determinant is social support. Lack of psychosocial and emotional support during pregnancy can result in depression, stress, and anxiety (Ghosh et al., 2010). Gestational violation is more likely in women experiencing these conditions. These violations not only lead to severe injuries to mothers but they may also induce preterm delivery, immature newborns, low birth weight and postpartum depression (Urquia et al., 2011).

With respect to the global importance of this condition and uncertainty of its etiology in 50% of cases and little attention from the public and research community (Queenan et al., Behrman and Butler, 2007), understanding effective factors of it is essential. In Iran, many studies have been conducted regarding the relationship of some social determinants of preterm delivery and pregnancy outcome. In these studies, some of the determinants such as age, socioeconomic condition, life style, anxiety, depression and unhealthy behaviors have been examined more concern. This study consolidated the findings of research in the last decade on preterm delivery in Iran in order to identify structural and intermediary determinants with respect to the World Health Organization (WHO) models to provide a perspective for other researchers in conducting future investigations.

## 2. Methods

A narrative review was performed.

### 2.1 Search Strategy

In this study, all Iranian articles from 2000 to 2010 were reviewed in 4 Persian databases including IranMedex (index of articles published in Iranian biomedical journals), SID (the Iranian Scientific Information Database), Irandoc (Iranian research institute for information science and technology and Magiran (the Scientific Magazines Bank of Iran) and 2 international databases including PubMed (database of United States National Library of Medicine) and Google Scholar. All of the scientific research journals of the Iranian medical universities were reviewed. Keywords of search were preterm delivery and pregnancy outcomes with (socioeconomic condition, anxiety, stress, depression, social support, violence, smoking, passive smoking, substance abuse, trauma and prenatal care during pregnancy).

### 2.2 Study Designs

All cross -sectional, longitudinal, cohort or case-control analytical designs published in peer- reviewed journals. Experimental and quasi – experimental designs were excluded.

### 2.3 Language

Articles in either English or Persian were considered for inclusion.

### 2.4 Inclusion Criteria

Studies were selected if the preterm delivery was reported and preterm delivery was defined as delivery between 20 to 37 weeks of gestation.

### 2.5 Study Selection

Accordingly, studies were selected in a two-stage process. First, the article were searched by title and then by the keywords [preterm delivery and outcome of pregnancy]. Two of the authors independently reviewed the titles and abstracts of the electronic database searches for any that appeared to match the inclusion criteria. The full text versions of any potentially relevant articles were obtained and reviewed by the same two authors using the inclusion criteria described above. Each of the authors compiled a list of articles to include that was compared, and any disagreements were resolved by consensus or arbitration. The sufficient reliability in data collection methods was assessed by a third person who had good knowledge of the subject but was blind to the names of the journal and the authors. The data presented in these studies were extracted, recorded in a table which was prepared for this purpose and analyzed using Excel, version 2006. Second, they were categorized with respect to WHO model (Figure 1) and social determinants affecting on health as follows:

- **Structural determinants** (Education, Occupation, Socioeconomic status)
- **Intermediary determinants** (Psychosocial factor [Stress, Anxiety, Social support, Types of pregnancy (Wanted or unwanted)] Behavioral factor and Maternal circumstance [Violence, Substance abuse, use of alcohol, Smoking, Passive smoking, Trauma, Place of residence] and Health system [Prenatal care])

### 2.6 Article Categorization

In this review, from 52 searched articles, 35 ones were selected for reading because of subject irrelevancy and duplication of citation in 8 databases. In some articles, a few determinants were studied collectively.

**Figure 2:**
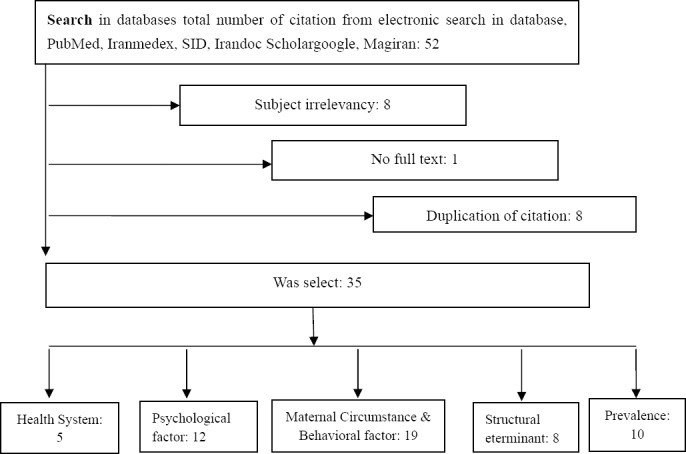
Study selection flow chart

## 3. Results

From 35 articles, 26 studies were descriptive (cross-sectional, longitudinal), 9 were analytical (cohort, case-control). 10 articles reported the prevalence of preterm delivery in different cities of Iran. The highest prevalence was in Kerman city (39.4%) and the lowest was in Quom (5.6%) (Table 1).

**Table 1:** Prevalence of preterm delivery in Iranian studies from 2000 to 2010

Author, Year, (Reference No.)	Study type	n	City	Prevalence
Jandaghi et al., 2011	Cross-Sectional	10913	Qom	5.6%
Afrakhteh et al., 2002-2003	Descriptive	5628	Tehran	7.2 %
Sohrabi and Ghanbari Gorkani, 2011	Cross-Sectional	3102	Zanjan	7%
Ganji et al., 2009	Descriptive	1237	Qom	13.9%
Mirzaie and Mohammah-Alizadeh, 2007	Descriptive	988	Kerman	39.4%
Sehati shaghaie et al., 2010	Descriptive	960	Ardabil	13.4%
Bayat Mokhtary et al., 2009	Descriptive	720	Mashhad	6.1%
Lotf Alizadeh et al., 2005	Case-Control	600	Mashhad	16.4%
Khakbazan et al., 2007	Cross-Sectional	518	Tehran	17.2%
Khalaji Nia and Sadeghi Moghaddam, 2011	Cross-Sectional	400	Qom	5.6%

Different factors related to preterm delivery were mentioned in the articles. Of them, with respect to the aim and inclusion criteria of the subjects based on WHO model (Solar & Irwin, 2010), variables including structural determinants (education, occupation, job experience and socioeconomic condition), behavioral factors, unhealthy behaviors, maternal circumstances (trauma; violence of the spouse; domestic violence; physical, sexual as well as emotional violence; smoking; alcohol consumption; drug abuse; place of residence; self-care; passive smoking), psychological factors (emotional stress, anxiety, type of pregnancy (wanted or unwanted), stress management and social support) and health system (prenatal care) were studied. These articles are summarized in terms of factor type in tables 2-5.

**Table 2:** Structural determinants related to preterm delivery in Iranian studies from 2000 to 2010

Author, Year, (Reference No.)	Study type	n	Risk Factor
Jandaghi et al., 2011	Cross-Sectional	10913	Occupation#
			Educational degree#
			Socioeconomic status•
Mirzaie and Mohammah-Alizadeh, 2007	Descriptive	988	Occupation#
			Educational degree#
Sehati shafaie et al., 2010	Descriptive	960	Occupation #
			Educational degree•
Lotf Alizadeh et al., 2005	Case-Control	600	Occupation •
			Socioeconomic status#
Khakbazan et al., 2007	Cross-Sectional	518	Occupation #
Khalaji Nia and Sadeghi Moghaddam, 2011	Cross-Sectional	400	Occupation #
Kamali Fard M, 2010	Case-Control	396	Occupation •
			Educational degree•
Amini and Savaie, 2011	Case-Control	244	Occupation #

• A significant relationship between this variable and preterm delivery was found in this investigations

# No significant relationship between this variable and preterm delivery was found in this investigations

**Table 3:** Psychological factors related to preterm delivery in studies from 2000 to 2010

Author, Year, (Reference No.)	Study type	n	Risk Factor
Jandaghi et al., 2011	Cross-Sectional	10913	Stress•
Ganji et al., 2009	Descriptive	1237	Stress #
Âbdollahy and Mohamadpor, 2004	Descriptive	1200	Types of pregnancy (wanted or unwanted) #
sehati shafaie et al., 2010	Descriptive	960	Stress #
			Types of pregnancy (wanted or unwanted •
Nasiri et al., 2009	Cohort	682	Anxiety•
Lotf Alizadeh et al., 2005	Case-Control	600	Stress•
Khalaji Nia and Sadeghi Moghaddam, 2011	Cross-Sectional	400	Stress•
			Types of pregnancy (wanted or unwanted)•
			Social support#
Kamali Fard M, 2010	Case-Control	396	Social support•
Ardekani and Salari, 2005	Case-Control	385	Stress•
Dabbaghi et al., 2001	Descriptive	360	Anxiety•
			Stress•
Shah Hosseini et al., 2008	Cohort	282	Anxiety•
Alipour et al., 2011	Cohort	156	Anxiety#

• A significant relationship between this variable and preterm delivery was found in this investigations

# No significant relationship between this variable and preterm delivery was found in this investigations

**Table 4:** behaviors factor and maternal circumstances related to preterm delivery in studies from2000 to 2010

Author, Year, (Reference No.)	Study type	n	Risk Factor
Mesdaghinia et al., 2009	Descriptive	5500	Trauma•
Faramarzi et al., 2005	Descriptive	3275	Violence•
Mirzaie and Mohammah-Alizadeh, 2007	Descriptive	988	Passive Smoking•
Sehati shafaie et al., 2010	Descriptive	960	Smoking #
			Place of residence # Substance abuse #
			Passive Smoking#
Khosravi and Hasheminasab, 2008	Cross-Sectional	870	Violence #
Hasheminasab, 2007	Cross-Sectional	840	Violence #
Negahban et al., 2010	Cross-Sectional	641	Passive Smoking•
Lotf Alizadeh et al., 2005	Case-Control	600	Smoking #
			Substance abuse #
			Place of residence #
			Trauma•
Delaram M, 2006	Case-Control	600	Passive Smoking•
Bodaghabadi, 2005	Cross-Sectional	587	Violence#
Dolatian et al., 2010	Descriptive	500	Violence•
Sharifian et al., 2011	Cross-Sectional	472	Substance abuse•
Mohammadian et al., 2000	Case-Control	450	Substance abuse•
Nojomi and Akrami, 2006	Descriptive	406	Violence•
Bagherzadeh et al., 2008	Descriptive	400	Violence •
Kamali Fard M, 2010	Case-Control	396	Smoking•
			Use of alcohol•
			Substance abuse•
Dolatian et al., 2001	Descriptive	104	Violence•
Ashrafganjoie et al., 2011	Cross-Sectional	80	Trauma•
Masoudi et al., 2008	Descriptive	71	Violence#

• A significant relationship between this variable and preterm delivery was found in this investigations

# No significant relationship between this variable and preterm delivery was found in this investigations

**Table 5:** Health system quality and preterm delivery in studies from 2000-2010

Author, Year, (Reference No.)	Study type	n	Risk Factor
Jandaghi et al., 2011	Cross-Sectional	10913	Prenatal care•
Sehati shafaie et al., 2010	Descriptive	960	Prenatal care#
Lotf Alizadeh et al., 2005	Case-Control	600	Prenatal care #
Khalaji Nia and Sadeghi Moghaddam, 2011	Cross-Sectional	400	Prenatal care•
Zafarghandi et al., 2004	Cross-Sectional	257	Prenatal care•

• A significant relationship between this variable and preterm delivery was found in this investigations

# No significant relationship between this variable and preterm delivery was found in this investigations

### 3.1 Structural Determinants

Employment of mothers was investigated in 8 studies showing no significant relationship between preterm delivery and working, in 6 studies and a significant relationship in the other 2. Maternal education was found to be related to preterm delivery in 2 studies and not related in 2. Socioeconomic condition was related to preterm delivery in 1 study and not related in the other (Table 2)

### 3.2 Intermediary Determinants

Psychological factors were investigated in 12 studies. Of them, emotional stress was referred to in 7 studies, 5 of which found a significant relationship between emotional stress including sensational stress as well as stressful events of life and preterm delivery. In addition, anxiety was studied in 4 investigations, 3 of which showed a significant relationship with preterm delivery. Type of pregnancy regardless of being wanted or unwanted was found to have a significant relationship with preterm delivery in 2 studies out of 4. Social support was found to have a significant relationship with preterm delivery in 1 study and no relationship in the other.

19 articles were about the relationship between behaviors factor of mothers and maternal circumstances and preterm delivery. Among these behaviors, domestic violence including physical, emotional and sexual had the most significant relationship with preterm delivery; among 9 related studies, 5 revealed the relationship in this regard. On the other hand, drug abuse and addiction of mothers showed the relationship more than other factors (5 articles). Smoking in 3 studies had a significant relationship with preterm delivery in 2 of them. Place of residence in 2 studies had no significant relationship. Passive smoking in 4 investigations showed a significant relationship with preterm delivery in 3 of them. Trauma to mothers during pregnancy in 3 studies revealed the significant relationship in all of them. (Table 4)

Prenatal care in health system including lack or irregularity of care was stated in 5 studies, which showed a significant relationship with preterm delivery. (Table 5)

## 4. Discussion

The studies reviewed here reported a prevalence of preterm delivery between 5.6% and 39.4% in Iran. Berghella (2010) reported the prevalence as 6% and noted that the distribution of preterm delivery differs between countries, as 85% of cases occur in Asia and Africa. Graafmans et al, suggested that differences in the prevalence of preterm delivery in developing countries might be due to a lack of reliable health databases and inconsistent methods of measuring gestational age, registering births, defining preterm delivery, diagnosing the viability of preterm infants, and burying the infants according to religious rituals, which can restrict the registration process (Graafmans et al., 2001).

Iranian researchers have investigated the social and structural determinants of preterm delivery in terms of occupation, education and socioeconomic status. Socioeconomic status is one of the most important determinants of health and mortality (Genereux et al., 2008) and is usually used to describe social inequalities. Some references used a combination of education, occupation, and income as a socioeconomic indicator. Pregnancy outcomes are strongly influenced by socioeconomic status; socioeconomic inequalities are an influential factor in the health of newborns and success of pregnancy outcomes, including preterm delivery (Berghella et al., 2007).

Women having low socioeconomic status and education levels are at double the risk of preterm delivery compared with women having high socioeconomic status and education levels. Educational inequalities due to inappropriate mixture of different gestational features, psychosocial factors, and lifestyle habits can be observed in women with lower education levels (Jansen et al., 2009). According to Shaikh et al. (2011), the higher the education levels of the father, the lower the risk of preterm birth. Education has been promoted as an important avenue for the improvement of socioeconomic status (Jansen et al., 2009).

Blumenshine et al, in a systematic review of articles suggested a strong influence of socioeconomic condition on the development of preterm delivery (Blumenshine et al., 2010). However, poor socioeconomic status is unlikely to have a direct and independent effect on preterm delivery; rather, the duration of pregnancy is shortened because of unhealthy behaviors, stress, and psychological reactions associated with stress (Kramer et al., 2001) In addition, a significant gap in health status may be observed between different regions in the country and between women of different social groups. Although not all these factors can be described as injustice, health inequality is evident. Wide disparities in income distribution and education may contribute to this gap in health status. Most Iranian studies included in this review considered occupation and education as demographic factors; however, socioeconomic inequalities were not always accurately measured.

Most Iranian studies found significant relationship between stress, anxiety, and preterm delivery. Investigations in other countries also showed significant relationships between preterm delivery and stressful life events, anxiety, depression, job stress, physical abuse, and weak social support (Kramer et al., 2001). Unhealthy behaviors related to ineffective coping mechanisms, such as smoking and drug abuse, are associated with preterm delivery. Low weight gain during pregnancy is also related to these behaviors, which are more prevalent in women who experience stress (Copper et al., 1996).

Stress acts through three mechanisms: the hypothalamic–pituitary–adrenal axis, activation of the inflammatory process, and ischemic mechanisms, all of which can be influential in inducing preterm delivery (Copper et al., 1996). In response to maternal emotional and physical stress, the endocrine system (i.e., the hypothalamic–pituitary–adrenal axis) is activated, releasing hormones such as adrenocorticotropin, cortisol, cytokine, and prostaglandins. On the other hand, the immune system activates the inflammatory process under conditions of physical stress. For example, infection triggers the release of neurochemical agents such as macrophages, endotoxins, cytokines, and prostaglandins, all of which can contribute to the development of preterm delivery (Strange et al., 2009). These mechanisms cause physiological responses and behavioral changes, including irritability, decreased food intake, anorexia, decreased sexual activity, increased depression and anxiety, and increased risk of violation (Latendresse, 2009).

A significant relationship between social support and preterm delivery was found in two investigations included in this review. In recent years, studies related to social support suggest that close, intimate relationships may have many positive and useful effects (Marmot and Wilkinson, 2006). A significant relationship has also been identified between social support and health. People with more social support have better health (Bovier et al., 2004, Berkman, 1995). In addition, physiological responses to stress are affected by social support; emotional reactions are less severe when a person is surrounded by relatives or friends (Glynn et al., 1999). Ghosh et al, (2010) indicated that lack of paternal support and chronic stress during gestation were possible risk factors for preterm delivery and that pregnancies for mothers with moderate or strong support had better outcomes (Ghosh et al., 2010).

Studies related to intermediary determinants such as unhealthy maternal behaviors and living circumstances most frequently addressed violation during pregnancy, smoking, and drug abuse as factors significantly related to preterm delivery. Studies show that violated women experience a higher level of stress and endocrine system reacts to it by releasing hormones. Thus, violation and its associated stress influence the endocrine and immune systems, which affect the development of preterm delivery and may lead to physical and psychological problems (Dolatian et al., 2010). In addition, Varma et al. (2006) stated that higher levels of depression is observed in violated women possibly because of the effects of violence on self-concept, especially in terms of self-confidence, dignity, and competence (Varma et al., 2007). The high prevalence of preterm delivery in some cities of Iran, for example, Kerman, may be due to intermediary determinants such as smoking or exposure to second-hand cigarette smoke, or Waterpipe (hookah) tobacco smoking in Pregnancy (Mirahmadizadeh & Nakhaee, 2008).

Physical and psychological symptoms, anxiety, inadequate care, and lack of social support may also occur in households where domestic violence occurs. These factors may have an impact on conception, attitude, tolerance, health, and well-being. In these households, the possibility of risky behaviors such as smoking and drinking is increased. Malnutrition and insufficient prenatal care in these settings may result in severe complications necessitating hospitalization. These factors inevitably affect the physical and mental condition of pregnant women, making it worse (Dolatian et al., 2010; Dolatian et al., 2012).

Smoking during pregnancy also has negative effects on maternal and infant health (Kathleen Adams et al., 2002). The resulting harmful health outcomes also impose a burden on the health system (Melvin et al., 2004). For example, babies of mothers who smoke are more likely to be born prematurely, have low birth weight, and die because of sudden infant death syndrome compared with those of healthy mothers (Khatiwada et al., 2010). As a result of smoking, certain biological mechanisms may be at work, including placental vasoconstriction and increased levels of catecholamines, which are initiators of labor (Crane et al., 2011). However, most smoking-related complications can be prevented through smoking cessation programs during and after pregnancy (Ayadi et al., 2006).

Some studies on the Iranian health system were also related to prenatal care. Significant relationships were found between absence, lack, or irregularity of prenatal care and preterm delivery in four out of six investigations. Prenatal care is known to have positive effects on maternal and infant health conditions (Liu, 1999). Health professionals have the opportunity to assess the health of the mother and the fetus, allowing time to prepare for interventions as necessary in order to prevent or minimize undesired health outcomes (Alexander and Korenbrot, 1995). In addition, regular checkups are beneficial in controlling or decreasing the incidence of gestational complications (Khatiwada et al., 2010).

## 5. Conclusion

In this review, despite methodological differences among studies in terms of type, sample size, ethnic group, and place, the results were very similar. Accessibility to full texts was limited for a few articles. However, the results show that preterm delivery is a common problem with critical complications in Iran as elsewhere. In many cases, appropriate interventions such as life skills trainings, self-care, and adequate prenatal care can prevent this outcome. In Iran, more research has been conducted on demographic factors and no study on the impact of economic inequality and incidence of preterm delivery has been found. In addition in many studies sample size was low and the method is not reliable, and no research has been done on the effects of structural with the intermediate social determinant and preterm delivery, then, it is suggested that studies is showed the impact inequality on incidence of preterm delivery should be designed and performing etiological investigations is recommended.
